# Instant pH sensor based on the functionalized cellulose for detecting strong acid leaks

**DOI:** 10.1098/rsos.211660

**Published:** 2022-03-16

**Authors:** Hoseong Jeong, Byung Jun Jung, Jae Hyun Kim, Seung-Ho Choi, Yoon Jung Lee, Kang Su Kim

**Affiliations:** ^1^ Department of Architectural Engineering and Smart City Interdisciplinary Major Program, University of Seoul, 163 Seoulsiripdaero, Dongdaemun-gu, Seoul 02504, Republic of Korea; ^2^ Department of Materials Science and Engineering, University of Seoul, 163 Seoulsiripdaero, Dongdaemun-gu, Seoul 02504, Republic of Korea; ^3^ Department of Architectural Engineering, University of Seoul, 163 Seoulsiripdaero, Dongdaemun-gu, Seoul 02504, Republic of Korea

**Keywords:** fibre optic sensor, pH sensor, covalent immobilization, cellulose, strong acids

## Abstract

Acid spills cause large-scale environmental damage and casualties. To respond to such incidents, a sensor capable of detecting acid leaks is required. Cellulose is a useful substrate material for the fast detection of acid leaks because it has high hydrophilicity and porosity. On the other hand, methods of manufacturing cellulose-based sensors are still complicated or time-consuming. Thus, in this study, a simple and rapid synthesis method for a cellulose-based pH sensor was proposed. The functionalization of α-cellulose was achieved via chloroacetyl chloride, and Congo red was covalently immobilized to the functionalized cellulose for detecting strong acids. The manufacturing process was composed of two steps as above and finished within 8 h. The developed sensor exhibited absorbance changes in the pH range of 0.2 to 3.0, and response time was shorter than 1 s. A prototype system using this sensor was manufactured and tested, and it detected acid leaks easily and quickly.

## 1. Introduction

Acids are fundamental reagents commonly used in chemical processes, and they are used as reaction catalysts [1–3], neutralizing agents [4,5], impurity treatment agents [6], rust removers and electrolytes in wet-cell batteries. Contrary to their usefulness, their strong corrosiveness and toxicity result in casualties, environmental pollution and failures of underground infrastructures [7]. To this day, acid spills still occur during transportation [8], storage and use in factories [9,10], and they cause heavy casualties and environmental damage [11,12].

Sensors capable of detecting acid leakage can be very useful for preventing or lowering such incidents. Acid leaks can be monitored by sensing pH, and the pH has been measured using electrochemical and nonelectrochemical methods [13–15]. A typical electrochemical method is to measure a potential difference between measurement and reference electrodes and convert the difference into pH by the Nernst equation [16]. Among the measurement electrodes, glass electrodes have various advantages, such as long-term stability, selectivity and slope. However, glass electrodes are vulnerable to physical impacts and are difficult to miniaturize and install flexibly [13].

A typical method of nonelectrochemical measurement is to use organic compounds that change their optical properties depending on the concentration of proton [13]. Although the organic compounds show a limited dynamic range (i.e. sensing range) and nonlinear response, they have excellent flexibility and impact resistance, which enable a more flexible installation [17]. In addition, by sending a signal through an optical fibre, it is possible to miniaturize a sensor system and avoid electromagnetic interference. Based on these advantages, numerous studies have been conducted on optical sensors over approximately 50 years [18–23], and this sensing mechanism is also used to detect silver [24], dysprosium [25], gadolinium [26] and lutetium [27].

Fabrication methods of sensing materials can be categorized into physical and chemical immobilization. The former is a method to physically entrap pH indicators into a porous substrate. Its advantages are that the manufacturing process is simple and the sensing range can be expanded by entrapping various pH indicators [28,29]. However, pH indicators are continuously leached because they are smaller than the pore size of a substrate [30]. To prevent leaching, the pore size can be reduced, but in this case, the responsibility of the sensor is greatly reduced [31]. The latter is a method that forms covalent bonds between a substrate and pH indicators, which makes a sensor resistant to leaching. Due to these covalent bonds, the high porosity of a substrate can be sufficiently secured, and as a result, the responsivity of a sensor can be improved. Based on these advantages, studies on covalent immobilization have been carried out, and sensors using cellulose derivatives as a substrate have attracted considerable attention because of their excellent responsivity as summarized in table 1.

**Table 1.** Previous research compared to this study.

researcher	functional group of dyes	immobilization		pH range	response time	application or purpose
		time^a^	step^a^			
Kostov [32]	amino group	52 h	4	1.3–4.0	30 s	biosensor
Ensafi & Kazemzadeh [31]	amino group	62 h	2	3.0–6.0	less than 5 s	not specified
Nawaz *et al.* [33]	amino group	16 h	2	1.0–2.0, 12.6–14.0	—	pH test strip
Werner & Wolfbeis [34]	sulfonyl group	1.5 h	4–5	10.0–13.0	50–60 s	alkali error correction
Mohr [35]	sulfonyl group			1.0–6.0	300–600 s	cellulose-based sensor layer
Ding *et al.* [36]	sulfonyl group			7.0–12.0	1 s	shrimp spoilage monitoring
this research	amino group	8 h	2	0.2–3.0	less than 1 s	acid leak detection

^a^The fabrication time and steps after deacetylation of cellulose acetate were counted.

Krysteva *et al*. [37] proposed a functionalization method of cellulose using urea and formaldehyde. Kostov *et al*. [32] formed covalent bonds between the functionalized cellulose and pH indicators which have aromatic amine. This process was composed of four steps and took 52 h. The absorbance of their sensor showed changes over the range of pH 1.3–4.0, and the response time of the sensor was about 30 s. Ensafi & Kazemzadeh [31] used thiourea and polyvinyl alcohol to form covalent bonds between cellulose and pH indicators. This process was composed of two steps and took 62 h. Their sensor showed absorbance changes over the range of pH 3.0–6.0 and a short response time within 5 s. Nawaz *et al*. [33] combined 1,10-phenanthroline-5-amine with cellulose acetate using 4,4′-methylene diphenyl diisocyana. Through this reaction, the sensor responding in both strong acid and alkali was produced by a two-step process within 16 h. Werner & Wolfbeis [34] introduced vinyl sulfonyl groups into pH indicators to form covalent bonds between cellulose and the pH indicators. Although this process required many reaction steps, all the reaction was finished within 1.5 h. Their sensor was able to detect alkaline substances, and its response time was 30 to 60 s. Mohr [35] immobilized his novel dye to cellulose acetate using this process, and his sensor showed the colour change in a wide acidic range. Ding *et al*. [36] also developed a novel dye by forming a diazonium coupling between 1-naphthol and 4-(ethylsulfurate sulfonyl)aniline and immobilized it onto cellulose using the method devised by Werner & Wolfbeis [34]. The sensor showed a very quick response within 1 s and was able to detect alkaline substances. As such, various covalent bonding methods have been proposed, but these methods are still complex or take a long time for manufacturing.

In this study, a relatively simple and fast method for manufacturing an acid detection sensor was proposed. Section 2 describes materials, apparatus and sensor fabrication methods. Section 3 presents the verification of the covalent bond, optical characteristics, responsibility, reversibility, effects of ionic strength, stability, thermal stability and effects of photoisomerization of the sensor. Section 4 shows application examples of proposed sensors. Section 5 summarizes the conclusions of this study.

## 2. Material and methods

### 2.1. Materials

In this study, Congo red (Duksan) was used as a pH indicator, while an α-cellulose membrane (No. 100; Hyundai Micro) was used as a substrate. Chloroacetyl chloride (purity ≥ 96%; Kanto Chemical) was used for the functionalization of cellulose. Chloroform (purity ≥ 96%) was used as a solvent. Standard buffer solutions were obtained from Daejung Chemicals & Materials, and they were used to calibrate the pH meter.

The universal buffer solution was prepared in the same manner as that described by Safavi & Bagheri [38]. Boric acid (Daejung), phosphoric acid (Daejung) and citric acid (Duksan) were added to deionized water at a concentration of 0.04 M. The final pH was adjusted with NaOH (1 M, Daejung) or HCl (1 M, Duksan).

The effect of ionic strength was investigated using water-soluble salts. Among them, NaCl, KCl, NH_4_Cl, MgCl_2_, CaCl_2_ and Na_2_CO_3_ were obtained from Daejung Chemicals and Materials, while BaCl_2_ and Na_2_SO_4_ were purchased from Junsei Chemical and Kanto Chemical, respectively.

### 2.2. Apparatus

The pH meter used for the pH measurement was PM3 of CAS. Vertex 70 of Bruker (FTIR) was used to confirm the covalent bond between Congo red and α-cellulose. Optical microscopy was used to identify the microstructure of the developed sensor (HT004 of Himaxtech). Ultraviolet-visible (UV–Vis) spectrophotometry was used to measure the absorbance of the sensor (Optizen pop of KLAB). Spectrometry was used to measure the pseudocolour of the sensor (Konica Minolta of CM2500). Thermal stability was investigated using Q50 of TA Instrument.

### 2.3. Covalent immobilization between Congo red and α-cellulose

**Scheme 1.**
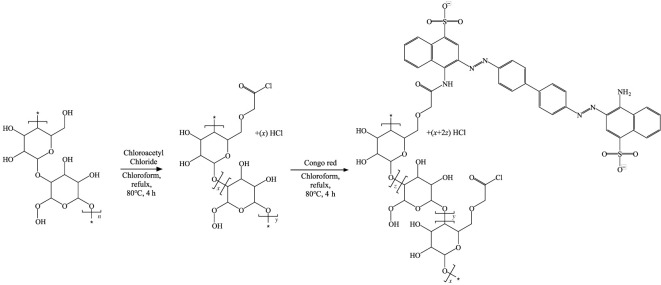
Covalent immobilization of Congo red onto α-cellulose.

Scheme 1 illustrates the covalent immobilization method proposed in this study. The cellulose was functionalized by a method of Adewuyi [39]. A small magnetic stirrer, chloroform (200 ml), cellulose (10 g) and chloroacetyl chloride (5 ml) were placed in a 500 ml flask, and then reflux and stirring were performed at 80°C for 4 h. In this process, acyl chloride was bonded to cellulose due to an SN2 reaction, and HCI was produced. In addition, the colour of cellulose gradually changed to dark green. After the first reaction step was completed, Congo red (0.1 g) was added, and then reflux and stirring were performed again at 80°C for 4 h. After the completion of the second reaction, Congo red immobilized cellulose (CRC), which is the reaction product, was placed in a teabag and washed with water and acetone to remove the remaining Congo red.

As presented above, the reaction time of the proposed method was 8 h, and the reaction step was two. Therefore, comparing the reaction time and steps of the proposed method to those of other methods shown in table 1, the proposed method is the fastest among the reactions using amino groups. On the other hand, the reaction is slower than the method using a sulfonyl group but simple.

## 3. Results

### 3.1. FTIR spectroscopy analysis

**Figure 1.**
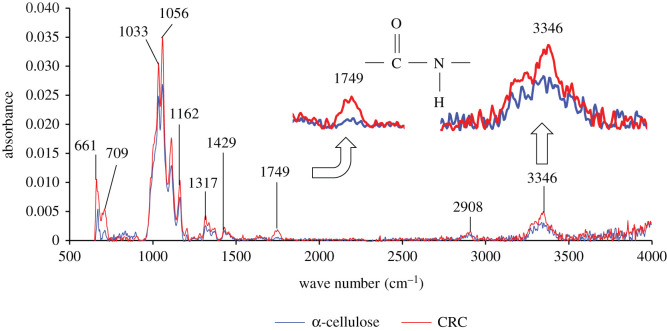
Comparison of FTIR results between the α-cellulose and CRC.

Figure 1 compares the FTIR results (transmission method) of α-cellulose and CRC. The *x*-axis and *y*-axis represent the wavenumber and absorbance, respectively, while a wavenumber of a peak is indicated above each peak. The broad peak at 3200–3550 cm^−1^ indicated the stretching of the O–H group of CRC and α-cellulose, while the peak at 3310–3360 cm^−1^ observed only for CRC suggested the stretching of the secondary amine. The peak at 2908 cm^−1^ represented C–H, which is present in both α-cellulose and CRC [40]. The peak at 1749 cm^−1^ suggested C=O introduced by chloroacetyl chloride. In addition, the peaks at 1429, 1317, 1162, 1056 and 1033 cm^−1^ for both α-cellulose and CRC indicated C–H, O–H, C–O, C–O and C–O bonds, respectively [40,41]. In short, the peaks at 3310–3360 cm^−1^ and 1749 cm^−1^ confirmed the amide bond between cellulose and Congo red.

Electronic supplementary material, figure S1(a) and S1(b) shows the images of α-cellulose and CRC obtained using optical microscopy. As shown in the electronic supplementary material, figure S1(a), the microstructure of α-cellulose was composed of porous fibre networks, and its colour was white. The CRC exhibited the same microstructure as that of α-cellulose, but its colour was orange due to immobilized Congo red, as shown in the electronic supplementary material, figure S1(b).

### 3.2. Optical characteristics of Congo red and Congo red immobilized cellulose

**Figure 2.**
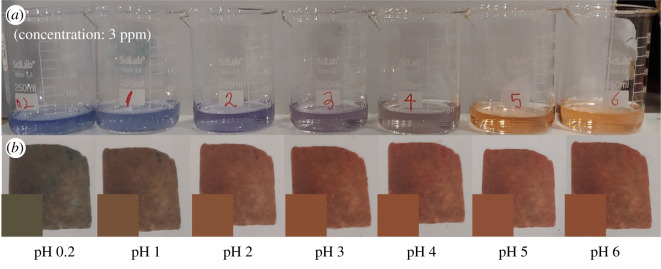
Colour change of Congo red (*a*) and CRC (*b*).

The colour changes of Congo red solutions and CRCs depending on pH are presented in figure 2*a*,*b*, respectively. The concentration of Congo red solutions was 3 ppm, and the pseudocolour of CRC was presented in the lower left corner of each CRC. In addition, the responses at higher pH levels are shown from left to right. As shown in the figure 2*a*, the Congo red solutions were blue at pH 0.2 to 2. At pH 3 to 4 and 5 to 6, the colours of the solutions were purple and orange, respectively. On the other hand, CRC was green at pH 0.2, brown at pH 1, light brown at pH 2, and orange at pH 3. There were no changes in colour at pH 3 to 6.

**Figure 3.**
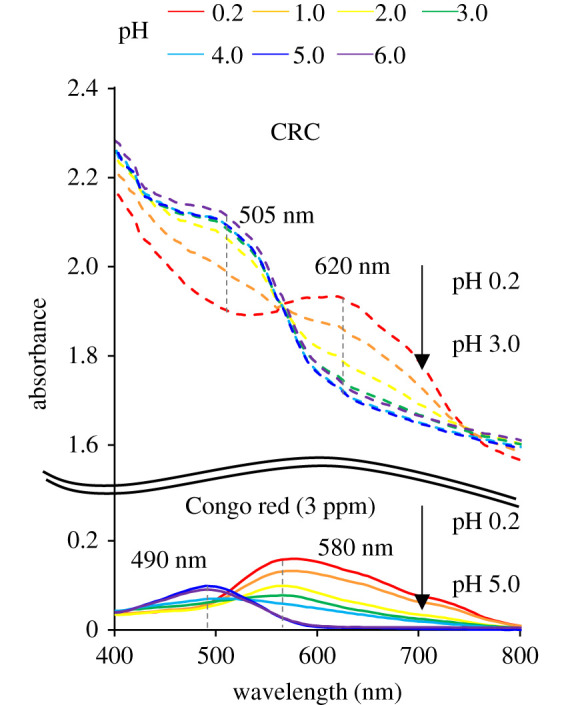
Absorbance spectra of the Congo red solution and CRC.

Figure 3 shows the absorbance spectra of the Congo red solutions and CRCs. The spectra of Congo red solutions and CRCs are indicated by solid and dotted lines, respectively, and each colour of the curve represents different pH environments. The *x*-axis and *y*-axis represent the wavelength and absorbance, respectively. As shown in figure 3, the maximum absorbance wavelength (*λ*_max_) of the Congo red solution was 580 nm at pH 0.2, and the absorbance at *λ*_max_ decreased with the increase in pH. In addition, as the pH increased, *λ*_max_ shifted to the left and became 490 nm at pH 5.0. On the other hand, *λ*_max_ of CRC was 620 nm at pH 0.2. Up to pH 3.0, the absorbance at 620 nm decreased, while the absorbance at 505 nm increased.

These spectra changes of Congo red and CRC are due to the protonation of basic functional groups, azo or amino groups. Pigorsch *et al*. [42] reported that two tautomers were generated from the protonation of Congo red at pH 5.3. As shown in electronic supplementary material, figure S2, one is ammonium form, where the proton is bonded to each amino nitrogen, and another is azonium form, where the proton is bonded to each α-azo nitrogen. By this protonation, Congo red becomes zwitterion. However, Yermiyahu *et al*. [43] discovered the cationization of Congo red at pH 1.2, based on which they concluded that an additional N atom was protonated at this stage.

### 3.3. Reversibility

**Figure 4.**
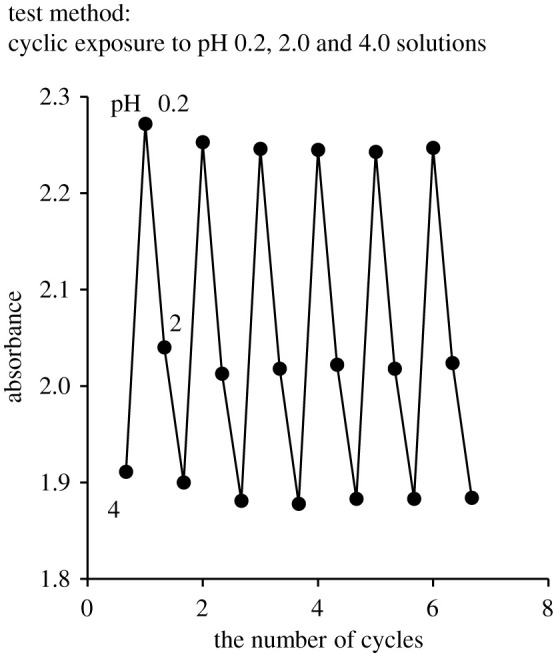
Reversibility of CRC.

A CRC was repeatedly immersed in solutions with pH 4, 0.2 and 2, in order, and its absorbance was observed to confirm the reversibility of CRC. Figure 4 shows reversibility test results of CRC, where the *x*-axis and *y*-axis represent the number of cycles and absorbance at 620 nm, respectively. The CRC exhibited almost uniform absorbance changes despite the cyclic exposures. This behaviour without leaching is attributed to the amide coupling between Congo red and α-cellulose, as shown in scheme 1.

### 3.4. Responsibility

**Figure 5.**
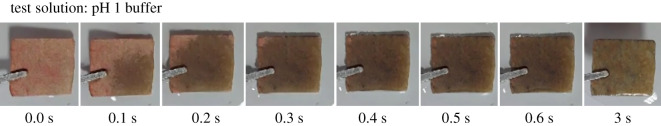
Responsibility of CRC.

The responsibility test was performed by dropping a CRC in a buffer solution with pH 1. The time required for the colour change of CRC was determined by image acquisition. Figure 5 shows the responsibility test results of CRC. The colour change of the CRC with time can be confirmed from the left side of the figure. As shown in the figure, the buffer solution penetrated the CRC at a very high speed. In addition, a rapid colour change within 1 s was observed by the sensor, which is a shorter response time than the previous sensors (table 1). This quick response is the most important characteristic of a leak detection sensor and is attributed to high porosity (electronic supplementary materials, figure S1) and multiple hydroxyl groups of CRC (scheme 1).

### 3.5. Ionic strength

According to Lewis & Randall, the ionic strength of a solution is defined as follows:

3.1 $$I=\frac{1}{2}\sum c_{i}z_{i}^{2},$$

where *c_i_* is the concentration of each ion (mole per litre) and *z_i_* is the charge of each ion. Since the ionic strength affects the activity of electrolytes including protons and the dissociation constant of the pH indicator [44], the colour of the pH sensor may be changed depending on the ionic strength as well as the pH of the solution. Therefore, related researchers have changed the ionic strength of a solution by modifying the type or concentration of salt and observed the absorbance difference of their sensor in the solution to determine the effect of ionic strength [45].

To investigate the influence of ionic strength on the spectral change of CRC, a salt-free solution and a salt solution of 0.02 M concentration were prepared. The pH of the solutions was 1.0, and the salts used in this experiment included NaCl, KCl, NH_4_Cl, MgCl_2_, BaCl_2_, CaCl_2_, Na_2_SO_4_ and Na_2_CO_3_. Absorption spectra of CRC were observed in both solutions, and the influence of external ions was calculated by

**Figure 6.**
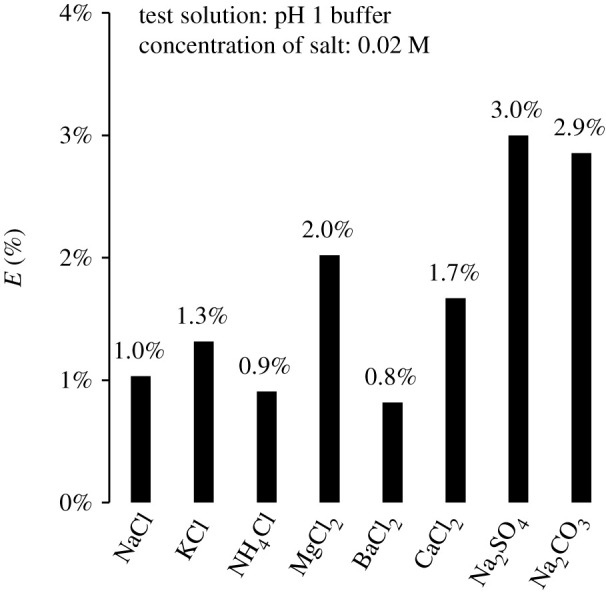
Effect of ionic strength.

3.2 $$E=\frac{\left|A_{a}-A_{0}\right|}{A_{0}}\times 100\;(\text{\%}),$$

where *A*_0_ is the absorbance at a wavelength of 620 nm of CRC exposed to the salt-free solution, and *A_a_* is the absorbance in the salt solution. Figure 6 shows the influence of each salt. The *x*-axis and *y*-axis represent the salt type and influence degree (*E*), respectively. The influence of ionic strength was smaller than 3%, and Na_2_SO_4_, Na_2_CO_3_ and MgCl_2_ exhibited higher degrees of influence than those of the other salts.

### 3.6. Stability

To identify the stability of the sensor, CRC was immersed in a pH 0.2 buffer solution for 91 days. Electronic supplementary material, figure S3 shows the absorbance spectra of CRC at pH 0.2, 2 and 4 after 0 and 91 days of exposure. As shown in the figure, even when CRC was immersed in the solution with pH 0.2 for 91 days, it exhibited almost the same absorbance spectra as that of the 0 days specimen.

### 3.7. Thermal stability

Because the thermal stability of materials is an important property for application [46,47], thermogravimetric analysis (TGA) was performed to determine the thermal stability of the CRC. Atmosphere gas was N_2_, and the heating rate was set to 10°C/min. Electronic supplementary material, figure S4 shows TGA curves of CRC and α-cellulose, in which the *x*-axis and *y*-axis represent temperature (°C) and weight (%), respectively. It is shown that the T_5%_, a temperature resulting in 5% weight loss based on initial weight, of α-cellulose was 317°C, but that of CRC was 270°C. This is because a part of the hydroxy group composing the secondary bond of cellulose was changed to Congo red which has less bond strength than the hydroxy group.

### 3.8. Effect of photoisomerization

Congo red has two azonium groups which are photoisomerizable. Thus, UV light can cause the photoisomerization of Congo red, and EE, EZ or ZZ isomer [48–50] occurs during this process. It was reported that Congo red in H_2_O exists as the EE isomer which has the lowest energy, and even when Congo red is turned into EZ or ZZ isomer, it returns to EE isomer within several hundred picoseconds [49]. Despite these results, this study investigated whether the photoisomerization of CRC is reversible. For this purpose, the absorbance spectra were observed for UV-exposed and non-exposed samples. The wavelength of UV light used to test was 200 nm, and the exposure time was 3 h. As shown in the electronic supplementary material, figure S5, the absorbance spectra of the CRC before and after the exposure were almost the same.

## 4. Application

### 4.1. Detection system

**Figure 7.**
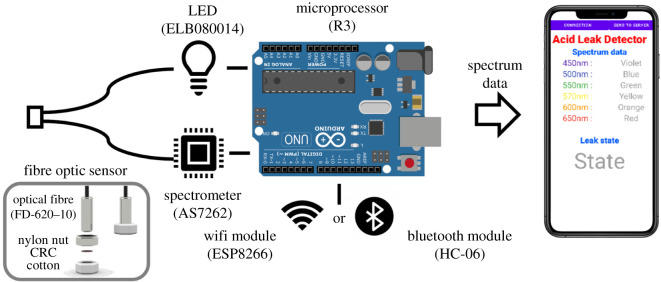
Detection system.

Figure 7 shows a system designed for the detection of strong acid leaks. A reflectance-based fibre optic sensor (FOS) is shown in the lower left of the figure, and the reflection distance between a CRC and an optical fibre is formed by an anti-loosening nylon nut (M6) assembled on an optical fibre (FD-620-10, Autonics). In addition, to fix CRC on the nylon nut, the nut and CRC were wrapped using cotton that can quickly absorb strong acids. The FOS was connected to an LED (ELB080014, ywrobot) and a spectrometer (AS7262, Adafruit) installed in a microprocessor (R3, Arduino), and data obtained from the spectrometer was transmitted to a smartphone through a wireless communication module. A smartphone app reads the data and determines whether an acid leak is occurring.

Electronic supplementary material, figure S6 represents reflectance data of CRC obtained by the prototype system to establish a leak criterion. As shown in the electronic supplementary material, figure S6(a), the ratio of reflectance at 650 nm to that of 600 nm reduced as pH decreased. These changes are shown in the electronic supplementary material, figure S6(b), and a leak criterion was set to the minimum value at pH 3 (i.e. 0.9).

### 4.2. Application

**Figure 8.**
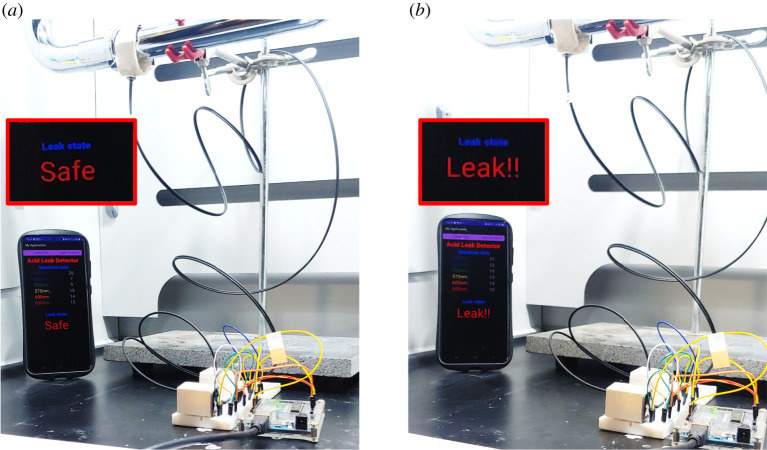
Application of detection system: (*a*) before leak and (*b*) after leak.

Figure 8 shows an application example of the prototype system. In the example, a strong acid leak in the pipeline was simulated, and the mobile app and detection system are shown on the left and right sides of the figure, respectively. When there was no leak, the ratio was higher than 0.9 and the app returned ‘safe’ as shown in figure 8*a*. On the other hand, if a leak occurred, the ratio dropped to less than 0.9, and the app returned ‘leak’ as shown in figure 8*b*.

The application of CRC is not limited to the detection of strong acid leaks in factories. This can be used to detect leaks in wet-cell batteries in automobiles and can also be used for smart packaging of acid bottles, chemical processes using a strong acid, environmental inspection and safety products.

## 5. Conclusion

A simple and rapid method for covalent immobilization between α-cellulose and indicator with the amino group was developed. The reaction time was relatively short, and the reaction process was simple compared to previous methods. The formation of covalent bonds was verified using FTIR spectroscopy, and an amide bond between cellulose and Congo red was observed. The developed sensor exhibited distinct changes in the absorbance spectrum at pH lower than 3 and excellent performances in terms of reversibility and stability. In particular, the developed sensor showed a rapid response (less than 1 s) suitable for acid leak detection. A prototype system and mobile app for detecting strong acid leaks were also proposed, and it was confirmed that acid leaks could be detected using them. As such, although the sensor developed in this study has various advantages for acid leak detection, there are several challenges to overcome in the future. One is the limited operating temperature of the developed sensor (less than 270°C), and another is environmental pollution due to Congo red used in manufacturing [51].

## Data Availability

The data and program codes can be downloaded from https://datadryad.org/stash/dataset/doi:10.5061/dryad.b5mkkwhf2.
